# Clinical application of right low-position modified peritoneal dialysis catheterization

**DOI:** 10.3892/etm.2012.808

**Published:** 2012-11-12

**Authors:** WEI REN, WEI CHEN, HUI-XUAN PAN, LEI LAN, PENG WANG, YE-HUA HUANG, MING KONG, YAN WANG

**Affiliations:** Renal Division, Anhui Provincial Hospital, Hefei, Anhui 230001, P.R. China

**Keywords:** peritoneal dialysis, catheterization, peritoneal dialysis catheter malposition

## Abstract

The aim of this study was to investigate peritoneal dialysis catheter malposition following low-position modified peritoneal dialysis catheterization and its clinical application value. A total of 48 patients receiving traditional peritoneal dialysis catheterization (the traditional group) and 95 patients receiving right low-position modified peritoneal dialysis catheterization (the modified group) from 2006 to 2011 were selected. The inflow time, outflow time, ultrafiltration volume of peritoneal dialysis solution and rate of peritoneal dialysis catheter malposition in the two groups of patients following surgery were compared and analyzed. There were no significant differences of inflow time, outflow time and ultrafiltration volume of the peritoneal dialysis solution between the two groups. In the modified group, no post-operative peritoneal dialysis catheter malposition occurred, therefore the incidence rate was 0. However, 9 patients in the traditional group presented peritoneal dialysis catheter malposition, an incidence rate of 18.75% (9/48). Among them, 6 patients required a second surgery. There was a statistically significant difference in the incidence rate of catheter malposition between the two groups (P<0.01). Right low-position modified peritoneal dialysis catheterization significantly reduced the incidence rate of peritoneal dialysis catheter malposition following peritoneal dialysis, and was shown to be significantly more effective than the traditional peritoneal dialysis catheterization and is therefore worth promoting for clinical use.

## Introduction

As a renal replacement therapy, peritoneal dialysis has been applied in the clinic since the 1970s. Compared with hemodialysis, it has a unique superiority since it requires only simple equipment and surgery and is more effective at protecting residual renal function, while having less impact on the internal environment of the body. In recent years, with significant improvement of the peritoneal dialysis connecting system and peritoneal dialysis solution biocompatibility, as well as increasing standardization of patients’ training, peritoneal dialysis has become the best method of early renal replacement therapy for patients with end-stage renal disease (1–4). For successful peritoneal dialysis, it is necessary to establish a persistent, effective and safe dialysis access. However, in current traditional peritoneal dialysis catheterization methods, a position at the left or right of the ventral midline, 10–12 cm above the pubic symphysis is usually selected for surgical incision (5). The peritoneal dialysis catheter is directed according to the guidelines into the Dow cavity (6). Therefore, the success rate of peritoneal dialysis catheterization remains unsatisfactory and the incidence rate of catheter-related complications is high. The inflow and outlet occlusion of the peritoneal dialysis solution caused by peritoneal dialysis catheter malposition is one of the more common complications (7). Also, a number of patients require further surgery, which creates additional problems for clinicians.

To resolve the problem of catheter malposition following traditional peritoneal dialysis catheterization, we used a swan-neck, straight catheter, placed into the right low position (surgical incision at 2 cm from the navel, 7–8 cm above the pubic symphysis, with the outlet on the right), to conduct peritoneal dialysis catheterization. Clinical observations confirm that no case presented peritoneal dialysis catheter malposition or inadequate drainage following peritoneal dialysis. This method greatly increases the rate of successful peritoneal dialysis catheterization.

## Materials and methods

### Clinical data

From 2006 to 2011, 48 patients receiving peritoneal dialysis using traditional peritoneal dialysis catheterization in our hospital were selected as the traditional group, including 19 female and 29 male patients. Their ages were between 21 and 73 years, and the average age was 42.6±13.5 years. In addition, 95 patients receiving peritoneal dialysis using right low-position modified peritoneal dialysis catheterization were selected as the modified group, including 36 female and 59 male patients. Their ages were between 16 and 85 years, and the average age was 47.6±17.0 years. This study was conducted in accordance with the declaration of Helsinki and with approval from the Ethics Committee of Anhui Provincial Hospital. Written informed consent was obtained from all participants.

### Preoperative evaluation

All participants were chronic renal insufficiency (CKD5) patients with dialysis indications and without peritoneal dialysis catheterization contraindications. Routine preoperative preparations were carried out (including preoperative catheterization and enema and one-time prophylactic application of antibiotic) 2 h prior to surgery.

### Incision selection

For the traditional group, surgical incision was performed 2 cm to the left of the navel, 10–12 cm above the pubic symphysis, and the outlet was on the left. For the modified group, the surgical incision was 2 cm to the right of the navel, 7–8 cm above the pubic symphysis, and the outlet was on the right.

### Surgical steps

Blood pressure, heart rate and blood oxygen saturation were monitored in the operating room and the drape was conventionally disinfected. The above positions were used as surgical incision points. Under local anaesthesia, a longitudinal incision with a length of 3–4 cm was made and subcutaneous tissues were separated to expose the anterior sheath of the rectus abdominis. Subsequently, the anterior sheath of the rectus abdominis was cut open to expose the rectus abdominis. Blunt separation of the rectus abdominis was carried out to expose the posterior sheath of the rectus abdominis. Then, the posterior sheath and peritoneum of the rectus abdominis were cut open to enable a swan-neck, straight catheter to pass. Under the direction of a steel guide wire, a swan-neck straight catheter was placed into the lowest part of the pelvic cavity (at the bladder-rectum fossa for the males and at the recto-uterine fossa for the females). To observe whether outflow liquid presented a streamlined flow, we injected water using a 50-ml syringe. The peritoneum was sutured with purse-string sutures, and the inner polyester cuff was outside the peritoneum. The anterior sheath of the rectus abdominis was sutured from the bottom to the top to seal the bottom of the inner polyester cuff, to allow for observation of possible seepage and leakage. Subcutaneous tunnel surgery was conducted using a subcutaneous tunnel needle in the same side to position the outer polyester cuff 2 cm away from the catheter skin outlet. Finally, the subcutaneous tissue and skin were closed and a postoperative abdominal bandage was applied.

### Postoperative care

Careful attention was paid to ensure that defecation was unobstructed following catheterization. If necessary, an appropriate amount of laxative was administered. After 24 h bed rest following catheterization, the patients were allowed to get up and exercise in moderation. In postoperative week 1, a 1.5% peritoneal dialysis solution was used for washing. If necessary, heparin was used for sealing the catheter. Subsequently, the intermittent peritoneal dialysis (IPD) mode was gradually changed to a non-bed peritoneal dialysis mode (CAPD).

### Observation indicators

We observed the inflow time, outflow time and ultrafiltration volume of the peritoneal dialysis solution for the two groups of patients in a follow-up visit. In addition, peritoneal dialysis catheter malposition incidence rates in the two groups were measured using abdominal radiography.

### Statistical method

SPSS 17 statistics software was used for analysis, and measurement data were expressed as the mean ± SD. One-way ANOVA was used for comparisons between groups. Sample rate comparison was carried out using the χ^2^ method. P<0.05 was considered to indicate a statistically significant difference.

## Results

### Clinical data

Peritoneal dialysis catheters used for all patients were swan-neck straight catheters provided by Baxter (Deerfield, IL, USA). The same surgeon carried out all procedures. For the 48 patients in the traditional group and the 95 patients in the modified group, the mean ages were 42.6±13.5 years and 47.6±17.0 years, respectively, and the male to female ratios in the two groups were 29:19 and 59:36, respectively. There were no statistically signficant differences between the two groups (P>0.05), including age, gender and underlying diseases. Among the patients in the traditional group, underlying diseases included 31 cases of chronic glomerulonephritis, 8 cases of diabetic nephropathy, 7 cases of hypertensive nephropathy, 1 case of lupus nephritis and 1 case of polycystic kidney. Among the patients in the modified group, underlying diseases included 73 cases of chronic glomerulonephritis, 10 cases of diabetic nephropathy, 8 cases of hypertensive nephropathy, 2 cases of lupus nephritis and 2 cases of polycystic kidney. Comparison of the number of underlying diseases between the two groups revealed no statistically signficant differences (P>0.05; Table I).

### Peritoneal dialysis

The dialysis mode was gradually changed from IPD to CAPD for all patients. After 1 week, the inflow and outflow speeds and ultrafiltration situations of the peritoneal dialysis solution were recorded continuously for 3 days at the same time point. There were no significant differences in the inflow time, outflow time and ultrafiltration volume of the peritoneal dialysis solution between the two groups (P>0.05). In addition, 1 patient presented abdominal pain near the bladder during peritoneal dialysis solution flow within the first postoperative month in the traditional group. In the modified group, 2 cases presented intermittent abdominal pain. Following peritoneal dialysis, the solution temperature was adjusted appropriately, the inflow and outflow rates were reduced and abdominal pain was relieved. One month after catheterization, the abdominal pain symptoms in these three patients had gradually disappeared.

### Peritoneal dialysis catheter malposition

As peritoneal dialysis catheter malposition mainly occurred within the month following catheterization (7), we carried out statistical analysis of peritoneal dialysis catheter malposition in all patients within 1 year of catheterization. In the traditional group, 9 cases presented postoperative poor drainage as well as peritoneal dialysis catheter malposition, confirmed by abdominal radiography, an incidence rate of 18.75%. Among them, 6 cases only required a second surgery, rather than a second catheterization. In the modified group, all cases presented postoperative poor drainage, and abdominal radiography confirmed that no peritoneal dialysis catheter malposition occurred, therefore the incidence rate was 0. There was a significant difference in the incidence rate of peritoneal dialysis catheter malposition between the two groups (P<0.01; Table II).

## Discussion

Accurate peritoneal dialysis access is the key to peritoneal dialysis success. At present, the incidence rate of peritoneal dialysis catheter-related complications is high, and the incidence of these complications is closely related to the peritoneal dialysis catheter insertion technique used, peritoneal dialysis center management and catheter design type (8,9). There are currently a number of types of peritoneal dialysis catheters that are applied in the clinic, including the Tenckhoff catheter, swan-neck catheter, Toronto Western Hospital (TWH) catheter, coil catheter and multiple-excellence type peritoneal dialysis catheter. Each type of catheter has different advantages and shortcomings due to their unique characteristics (10–13). The majority of retrospective studies indicate that the malposition rate of a coil catheter for Tenckhoff peritoneal dialysis is higher than that of a straight catheter. Certain studies, however, claimed that there was no significant difference between the two (14) or that the malposition rate of a coil catheter was less than that of a straight catheter (10). The malposition rate in shift catheters has been shown to reach 50% (15,16).

Current peritoneal dialysis catheterization techniques include percutaneous puncture catheterization, traditional surgical catheterization and laparoscope catheterization (17). Percutaneous puncture catheterization easily damages abdominal organs and may easily cause leakage of early dialysis solution, catheter malposition, drainage failure, infection and other complications. The laparoscope catheterization surgery is more complex and its cost is higher. Traditional surgical catheterization is the most commonly used method in China. However, domestic and international studies have shown that the incidence rate of catheter malposition for this method is between 15 and 20% (2), and a number of patients require a second surgery.

To resolve the problem of a high incidence rate of catheter malposition following traditional catheterization, we used right low-position modified peritoneal dialysis catheterization to place a swan-neck, straight catheter into the lowest part of the pelvic cavity (at the bladder-rectum fossa for the males and at the recto-uterine fossa for the females) and compared it with the homochronous traditional surgery. We found that there were no significant differences between the two groups for inflow time, outflow time and ultrafiltration volume of peritoneal dialysis solution. In the traditional group, 9 patients presented peritoneal dialysis catheter malposition, an incidence rate of 18.75% (9/48). Among them, 6 cases required a second surgery. In the modified group, no peritoneal dialysis catheter malposition occurred following catheterization, therefore the incidence rate was 0. Statistical analysis results showed that the modified operation effectively prevented the occurrence of peritoneal dialysis catheter malpositioning.

The possible explanations for this are: i) Omental wrapping is an important cause of peritoneal catheter malfunction. Omental wrapping frequently leads to catheter migration and progressive outflow failure. The low position (7–8 cm above the pubic symphysis) is in the lower third of the abdominal cavity and prevents the recurrence of omental wrapping; ii) A swan-neck, straight catheter was used, and the subcutaneous tunnel of the outer catheter goes downwards with the natural curvature of the body. This means there is a decrease in the incidence rate of peritoneal dialysis catheter malposition caused by the elastic tension of the peritoneal dialysis catheter (18); iii) Selection of the correct catheterization technique is important. If peritoneal dialysis catheter malposition occurs on the left of the abdominal cavity, as this is at the descending colon, normal downward peristalsis of the descending colon may reset the peritoneal dialysis catheter into the pelvic cavity. However, peritoneal dialysis catheter malposition mostly occurs on the right in the traditional left catheterization, as this is at the ascending colon. With this placing, it is very difficult to reset the peritoneal dialysis catheter into the pelvic cavity due to intestinal tympanites and the normal antral peristalsis of the ascending colon. Therefore, right low-position modified peritoneal dialysis catheterization increases the success rate of peritoneal dialysis catheterization, and reduces postoperative common peritoneal dialysis catheter malposition and the incidence rate of poor drainage. Additionally, it reduces postoperative pain in patients and improves peritoneal dialysis quality. This may benefit uremic patients and improve their quality of life.

As right low-position modified peritoneal dialysis catheterization only requires simple surgery, does not increase the pain and the economic burden for patients and avoids the occurrence of peritoneal dialysis catheter malposition and poor drainage following catheterization, it is worth promoting in clinical use. However, a large number of clinical randomized controlled trials of this technique are required for further verification.

## Figures and Tables

**Table I.** Basic data of the two groups.

	Traditional group	Modified group
Number of patients	48	95
Gender ratio (male: female)	29:19	59:36
Age (years)	42.6±13.5	47.6±17.0
Chronic glomerulonephritis	31	73
Diabetic nephropathy	8	10
Hypertensive nephropathy	7	8
Lupus nephritis	1	2
Polycystic kidney	1	2

For peritoneal dialysis catheterization, the swan-neck catheter was used. The surgeries were performed by the same person. All P>0.05 vs. the traditional group.

**Table II.** Incidence rate of peritoneal dialysis catheter malposition in the two groups.

Group	Cases	Catheter displacement (number)	No catheter displacement (number)	Catheter displacement rate (%)
Traditional group	48	9	39	18.75
Modified group	95	0^a^	95	0^a^

a P<0.01 vs. the traditional group.
